# Following the Action of Atypical Antipsychotic Clozapine and Possible Prediction of Treatment Response in Schizophrenia

**DOI:** 10.3390/life15060830

**Published:** 2025-05-22

**Authors:** Mihai-Gabriel Năstase, Antonia Ioana Vasile, Arina Cipriana Pietreanu, Simona Trifu

**Affiliations:** 1 Doctoral School, “Carol Davila” University of Medicine and Pharmacy, 020021 Bucharest, Romania; nastasemihaigabriel@yahoo.com (M.-G.N.); antoniaioana97.vasile@gmail.com (A.I.V.); arinapietreanu@gmail.com (A.C.P.); 2 Department of Neurosciences, “Carol Davila” University of Medicine and Pharmacy, 020021 Bucharest, Romania

**Keywords:** depression, neuroplasticity, receptor, gene

## Abstract

We tried to synthesize the possibilities of predicting the response to clozapine treatment, which can significantly improve the efficacy of the active substance and reduce adverse reactions, and how the active substance acts at the D1 dopaminergic receptors D2, D3, D4, and D5, muscarinic M1, M2, M3, and M5, and the histamine and alpha 1 adrenergic receptor, as well as how it contributes to increased cerebral blood flow, the effect on ribosomal protein S6 function, or the effect on kynurenine 3-monooxygenase function. Clozapine is one of the most effective antipsychotics, and there is potential to improve performance by combining it with different compounds to limit adverse effects or by augmenting it with other antipsychotics (amisulpride, paliperidone), other active substances with different properties (minocycline, N-acetylcysteine, memantine), or alternative therapies (electroconvulsive therapy, repetitive transcranial magnetic stimulation). There are also significant steps in optimizing clozapine efficacy by predicting treatment response, which could be determined by testing the following: plasma levels of clozapine N-oxide and N-desmethylclozapine, serum levels of neurotrophins and glutamate, genetic testing, the polygenic risk score, morphometry, or even the identification and accurate determination of persistent negative symptoms.

## 1. Introduction

There are substantial limitations in antipsychotic medication, which are due, on the one hand, to the as yet unexplained nature of how schizophrenia is triggered and affects the patient’s brain, and on the other hand, to the precise understanding of the mechanism by which antipsychotics act on the brain. This paper aims to bring together in one place information from the last 10 years of clinical research in an attempt to synthesize the latest information about the mechanisms of action of the atypical antipsychotic clozapine in the schizophrenic brain.

In order to arrive at an appropriate pharmacological intervention and to predict early response to treatment with different active substances, a better understanding of how they affect schizophrenia deficits is needed.

Knowing as much as possible about the mechanism of action and the limitations of active substances can help to develop new treatment strategies for patients with schizophrenia.

Antipsychotic medication focuses mainly on dopaminergic, serotonergic, and GABAergic hypotheses [1,2]. To a large extent, the main action of antipsychotics is closely related to dopaminergic receptors [3]. If we take into account patient outcomes following the administration of active substances and long-term effects, all these hypotheses are only partially supported. This is also one of the reasons why new-generation antipsychotics have been and are still being developed. There is a need for a broader overview of the action of antipsychotics in the brain. Recent research [4] brings, again with evidence, into question the possibility of parallel information transmission networks. Unlike mice and macaques, which use a single polysynaptic pathway for information transmission, which is also the focus of current research, the human brain also uses several secondary pathways to transmit information to other brain regions. In other words, in the human brain, information is also transmitted via alternative routes. Thus, we see that there are areas and functions that we have not previously identified and that offer new insights into understanding the hypercomplex system behind mental disorders. An in-depth study of this phenomenon could change the paradigm for the development of new treatment schemes and even new antipsychotics.

The ground for developing new ways to treat patients with schizophrenia is fertile. But until future research proves to be game changers, it is necessary to understand as much as possible about how the antipsychotics currently available in psychiatric hospitals work. In this paper, we will focus on what we currently know about how the atypical antipsychotic clozapine works.

## 2. Materials and Methods

Scientific articles of clinical trials were searched in three databases, namely Scopus, PubMed, and ClinicalTrials.gov.

The Scopus database was searched using the following keywords: “clozapine AND function AND brain” and selecting filters for the period from 2013 to 2025; the subject area being limited to pharmacology, toxicology, and pharmaceutics; the document type being limited to articles; keywords being limited to clozapine, schizophrenia, and human; the language being limited to English; and the research being limited to open access. A total of 30 scientific articles were identified, but a manual screening step was also performed to identify research only on human subjects diagnosed with schizophrenia on clozapine treatment. Following the analysis, 5 articles were identified that met the criteria.

The PubMed database search was conducted using the following keywords: “clozapine function in the brain”. Filters were used for clinical trials from 2013 to 2025. The search identified 16 scientific articles from clinical research involving human subjects diagnosed with schizophrenia and on clozapine treatment.

The ClinicalTrials.gov database was searched using the following filters: condition: schizophrenia; other terms: clozapine; study status: completed; eligibility criteria—sex: All; age: Adult; study results: with results; and date range from 1 January 2013 to 31 December 2024. We found 33 studies as a result.

Our team eliminated duplicate information and established a presentation results tree. Finally, we decided to add scientific information from several reviews and animal trials that we found significant for this research by searching the relevant terms in the PubMed database using the filter for last 5 and the last 10 years.

## 3. Results

### 3.1. Details of Clozapine Action in the Brain Affected by Schizophrenia

Unlike classical antipsychotics, clozapine acts as a dopaminergic and serotonergic receptor antagonist. It influences the dopamine D4 receptor and binds less to the D2 receptor, which is generally the target of classical antipsychotics. The active substance also antagonizes the muscarinic receptors of the parasympathetic system M1, M2, M3, and M5, the histamine receptors, and the alpha 1 adrenergic receptor [5].

Specifically, it acts by transiently occupying dopamine D2 receptors in the striatum, unlike other antipsychotics that have a prolonged occupation of these receptors [6]. The chemical structure of clozapine allows for a relatively rapid dissociation from D2 receptors, translated by the ability to rapidly dissociate or release from D2 dopamine receptors after its effect.

Clozapine interferes with dopamine [7] by binding to several of its main receptors, namely D1, D2, D3, and D5. These receptors are involved in regulating motor functions and other cognitive processes. It also has an increased affinity for the dopamine D4 receptor, which is associated with cognitive and behavioural functions. The substance exhibits an anticholinergic effect, interfering with the action of the neurotransmitter acetylcholine. It may impact cognitive function and contribute to anticholinergic side effects.

Because it acts [8,9] on several categories of dopamine, serotonin, glutamate, and N-acetylaspartate receptors, clozapine has increased efficacy and is considered [10] the gold standard in the treatment of treatment-resistant schizophrenia patients.

### 3.2. Increased Cerebral Blood Flow

A recent study [11] comparing cerebral blood flow in patients with schizophrenia treated with clozapine and patients treated with electroconvulsive therapy finds in SPECT CT scans a higher blood flow in certain brain regions (left prefrontal cortex LPFC and right temporal cortex RTC) following electroconvulsive therapy. However, increases were recorded in left hemisphere regions such as the left lateral prefrontal cortex (LLPFC) but not in the left temporal cortex (LTC). Partial increases were recorded in the right hemisphere regions, with the right medial prefrontal cortex (RMPFC) and right temporal cortex (RTC). Although the difference between clozapine and electroconvulsive therapy is significant, administration of the active substance in patients with schizophrenia contributes to increased cerebral blood flow.

The increase in blood flow brought about by clozapine treatment was also associated with improvements in negative and positive symptoms.

### 3.3. Hypofunction of Ribosomal Protein S6

One of the most recent findings that may underlie the mechanism of schizophrenia development is the hypofunction of ribosomal protein S6 in the prefrontal cortex of patients with schizophrenia [12], which is an effector of the mTOR pathway and plays an important role in regulating protein synthesis. When mTOR is activated, the ribosomal S6 protein is phosphorylated and participates in the control of protein translation. The importance of the mTOR pathway is crucial for the regulation of cell growth and proliferation. It interacts with Akt (protein kinase B), a key protein in the PI3K/Akt/mTOR signalling pathway, with a crucial role in regulating cell survival, neuronal growth and proliferation, and GSK3β (glycogen synthase kinase 3 beta), which is a protein involved in various cell signalling pathways. Within the PI3K/Akt pathway, GSK3β is inhibited by phosphorylation, impacting several cellular processes, including cell survival and development.

Clozapine, unlike risperidone, has a regulatory effect on Akt but not on GSK3β—in rat cortical tissue chronically exposed to antipsychotics, clozapine (but not risperidone) increased phospho-Akt(Ser473) without altering phospho-S6, whereas risperidone left Akt unchanged; neither compound produced a significant change in phospho-GSK-3β(Ser9) under the same conditions [12]. The active substance appears to decrease S6K phosphorylation in both the cortex and striatum; however, the data are not sufficient, and further research into this phenomenon is needed. In the rat frontal cortex, acute clozapine triggers a brief surge in p90RSK phosphorylation that peaks within the first hour and is followed, 60–120 min post-dose, by a pronounced fall below baseline—an overall pattern indicating a net downregulation of S6-kinase signalling [13]. An independent mouse study that monitored the same ERK → p90RSK cascade in both the cortex and striatum replicated this biphasic profile: an early activation phase gave way to a sustained reduction in p90RSK phosphorylation, again pointing to late-phase suppression of S6-kinase activity after clozapine exposure [14].

### 3.4. Inhibition of Kynurenine 3-Monooxygenase (KMO)

Schizophrenia impairs the ability of kynurenine 3-monooxygenase (KMO) to function by inhibiting it [15]. KMO inhibition, in turn, leads to the accumulation of kynurenine instead of being converted to 3-hydroxykynurenine, a key step in the kynurenine pathway. This produces more kynurenic acid (KYNA), which is the metabolite of kynurenine. KYNA acts as an antagonist of NMDA (N-methyl-D-aspartate receptor) and α7 nicotinic receptors. High concentrations of kynurenic acid (KYNA) block the glycine site on the NMDA receptor (N-methyl-D-aspartate receptor) [16]. As a result of this complex process, a significant increase in the spontaneous activity of dopaminergic (DA) neurons in the ventral tegmental area (VTA) has been observed.

Clozapine has an NMDA receptor agonist effect, and its activation, despite blockade of the glycine site by KYNA, could lead to the inhibition of dopaminergic (DA) triggering in the VTA.

### 3.5. Neurobiology of Clozapine-Resistant Patients

Clozapine is well known for its positive characteristics in treating patients resistant to treatment with classical antipsychotics and is considered a gold standard treatment. However, there is also a ⅓ to ½ category of patients resistant to clozapine treatment [17]. The latter are characterized by a profile of marked impulsivity and aggressiveness [18]. The most recent finding [19] identifies the right inferior frontal gyrus (rIFG) region that plays a key role in modulating the impulsive response.

Trying to draw a parallel, we could say that clozapine treatment could have an impact on rIFG. The claim is also doubled by the images resulting from neuroimaging investigations that measured cerebral blood flow after clozapine administration [11], showing an increase in this flow in the rIFG area. Thus, the need for more studies in this direction is noted.

Among treatment-resistant patients, there have been cases of complete remission of schizophrenia [20]. Imaging studies after clozapine administration for treatment resistance have shown novel results marked by increased grey matter volume in the cingulate cortex, parietal cortex, and subthalamic region.

Despite the results, few studies address the issue of total remission in schizophrenia. Although the percentage of recovered patients is unknown, it should be stressed that such cases are extremely rare but should be investigated in detail to gain new insights into how the recovery of schizophrenia-affected structures occurs.

In Table 1, we have summarized the information presented previously.

**Figure 1:**
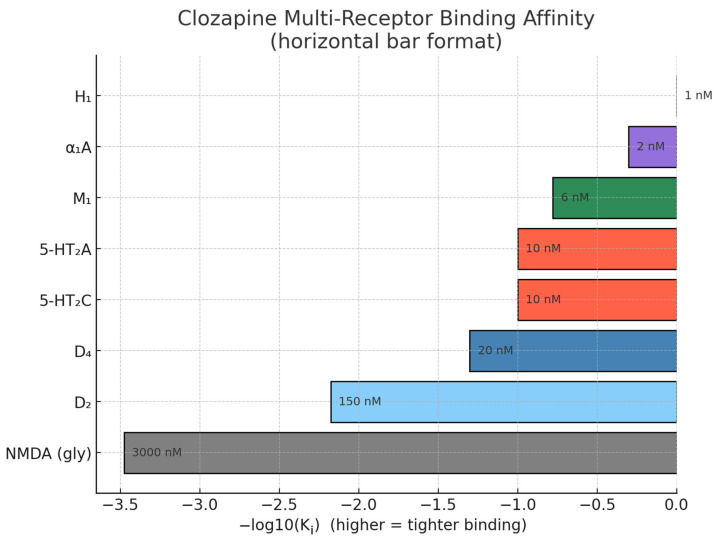
Clozapine multi-receptor binding affinity.

Clozapine displays a wide-ranging receptor-binding profile, acting as a generally low-potency antagonist at most sites while demonstrating exceptionally high affinity at a limited subset of specific receptors. A re-analysis of binding data from >30 studies confirmed that clozapine’s K_i_ for 5-HT_2_A is 5–10 nM—roughly an order of magnitude higher than its D2 affinity [18]. The 2017 “pharmareceptomic” atlas likewise placed 5-HT_2_A (pre-frontal cortex) and 5-HT_2_C (caudate) among the top outlier targets that differentiate clozapine from typical antipsychotics [19]. StatPearls’ 2023 pharmacology update reiterates that clozapine binds D4 with markedly higher affinity (≈20 nM) than D2 (≈150 nM) and occupies only 40–60% of striatal D_2_ sites at therapeutic plasma levels—well below the 80% threshold associated with extrapyramidal toxicity [20]. The Cardozo atlas highlights D4 in the pineal gland as one of the few clozapine-specific hotspots [19]. The same 2017 dataset reported K_i_ ≈ 6 nM for M_1_ (and 15–40 nM for M_2_–M_5_) [19]. The 2023 MDPI review corroborates strong antagonism at M_1_–M_3_ and notes that norclozapine partially agonizes M_1_/M_4_, a property now exploited by KarXT-style drug design [21]. Clozapine shows nanomolar H_1_ affinity (~1 nM) and equally tight binding at α_1_A adrenoceptors (~1–2 nM)—the highest of any clinically used antipsychotic [19]. In Figure 1 we present this multi-receptor binding affinity of clozapine. Occupancy at these sites predicts dose-related sedation and orthostatic hypotension and is the reason slow nocturnal titration is recommended [20]. Direct radioligand work is scarce, but recent translational reviews argue that clozapine acts as a partial agonist at the glycine modulatory site of NMDA receptors with affinities in the low-µM range, thereby enhancing NMDA throughput when endogenous glycine/D-serine is low [22]. This mechanism is increasingly invoked to explain clozapine’s unique pro-cognitive and anti-suicidal effects despite its weak D2 blockade. This spectrum explains why clozapine can deliver robust antipsychotic efficacy while minimizing motor side-effects (low D2), yet it carries risks of weight gain, sedation, and hypotension (H_1_, M_1_, α_1_A). It also underlines why changes in cholinergic or serotonergic tone, rather than dopaminergic occupancy alone, often dictate clinical response [23].

### 3.6. Treatment of Clozapine-Resistant Patients

A meta-analysis that pooled 7546 TRS patients from 77 trials with an overall response rate of 41%, with the highest baseline positive-symptom burden as the only patient variable that significantly predicted response, demonstrates that easily measurable baseline variables—age, sex, illness duration, and cognition—do not separate responders from non-responders. Positive-symptom severity only offers a coarse guide [24].

#### 3.6.1. Augmentation of Clozapine with Other Antipsychotics

In a multicenter, retrospective study [25], 4284 discharged patients prescribed clozapine as an active substance were analyzed. Of these, only 32.3% required clozapine augmentation. Of these, 51.4% received augmentation with atypical antipsychotics (SGA) and 48.6% had typical antipsychotics (FGA) as augmentation treatment. The same study also notes an underutilization of clozapine in psychiatric practice.

##### Augmentation with Amisulpride

For such cases, several treatment regimens have been studied to serve as an adjunct to clozapine. Recent studies include the administration of amisulpride in combination with clozapine [26,27,28]. The results show promise, with improvements in negative and cognitive symptomatology. However, there is a cost that seems quite serious. This research also points to a number of severe adverse effects, even cardiac, that amisulpride causes. It is important to study the administration of the active substances in detail to observe the long-term effects.

##### Augmentation with Paliperidone

Paliperidone, in combination with clozapine, works well in clozapine-resistant patients [29]. Research has shown augmentation treatment to be safe, reducing adverse effects unlike those caused by amisulpride, and effective in patients with treatment-resistant schizophrenia. Again, safety investigations are indicated, and the need for more randomized trials to establish the exact immediate and long-term effects is stressed.

#### 3.6.2. Augmentation of Clozapine with Anti-Inflammatory Medication

##### Minocycline

This is an antibiotic of the tetracycline class, used to treat various infections, especially those of a bacterial nature. It is studied [30] in the augmentation of clozapine for its anti-inflammatory and neuroprotective effects.

Clozapine augmentation with minocycline was determined by the high concentration of C-reactive protein, as an indicator of inflammation, obtained from peripheral blood. The results are promising, indicating a good tolerance of the active substance, without significant adverse effects, and a reduction in brain inflammation. However, the small sample of patients on which the treatment regimen was administered is not relevant to the general population.

The use of active substances with anti-inflammatory properties in schizophrenia is not new. There is much discussion regarding the inflammatory nature of schizophrenia, and the development of new antipsychotic treatments has included the anti-inflammatory effect [31].

Given the existence of a superior antipsychotic in treating patients with treatment-resistant schizophrenia, such as clozapine, augmenting the treatment regimen with antipsychotics could be key to increasing therapeutic efficacy and reducing potential adverse effects caused by other antipsychotics.

#### 3.6.3. Augmentation of Clozapine with Other Active Substances

##### N-Acetylcysteine (NAC)

This is a compound that can be converted to cysteine in the body. This can support the production of glutathione (GSH), an important antioxidant in the body, playing an essential role in protecting cells against oxidative stress. N-Acetylcysteine (NAC) may also have its own antioxidant and anti-inflammatory properties. It has been proposed for clozapine augmentation, but results have not identified significant improvements in symptoms produced by schizophrenia [32].

##### Memantine

This is an active substance used mainly for the treatment of memory disorders, having effects on glutamate neurotransmission by blocking N-Methyl-D-Aspartate (NMDA) receptors. It can modulate neuronal excitability.

Memantine is being studied in combination with clozapine for potential improvement in memory function, executive functions, and positive and negative symptoms in schizophrenia. In this case, too, the results are promising, with minimal adverse effects. Improvements occur in visual and verbal memory, as well as in terms of negative symptoms [33]. It has also been shown to be effective in treatment-resistant patients [34].

#### 3.6.4. Alternative Therapies in Clozapine Augmentation

##### Repetitive Transcranial Magnetic Stimulation (rTMS)

In addition to pharmacological adjuvants, there are new, innovative therapies such as repetitive transcranial magnetic stimulation, which has been studied together with clozapine [35]. The results of the study showed an increase in effectiveness in stopping psychotic productivity altogether. It is a new technique that could enhance the effects of psychiatric medication, but as this technology is in its infancy, more research is needed.

##### Electroconvulsive Therapy (ECT)

Electroconvulsive therapy has also been studied to enhance the efficacy of clozapine, especially for patients with treatment-resistant schizophrenia [36]. This has been shown to be effective, at least in the short term. It is safe and without significant adverse effects. There is a need for long-term research to investigate the persistence of the therapeutic effect of electroconvulsive therapy.

The next table (Table 2) describes the strategies to treat treatment-resistant schizophrenia.

### 3.7. Effect of Clozapine on Cannabis Use in Schizophrenia

A common problem among patients with schizophrenia is the use of cannabis and thus the comorbidity of the diagnosis of cannabis use disorder (CUD). Research results [37,38,39] aimed at reducing marijuana use in schizophrenia have revealed a significant effect of clozapine on reducing cannabis use among patients with schizophrenia. Further research in this direction is important to optimize the effects of antipsychotics for patients with co-morbid cannabis use disorder.

### 3.8. Possible Methods for Predicting Effectiveness of Antipsychotic Treatment

#### 3.8.1. Plasma Levels of Clozapine N-Oxide (CNO) and N-Desmethylclozapine (NDMC)

The time course of clozapine response was correlated with plasma levels of the active substance and its metabolites. Thus, treatment-resistant patients had significantly lower plasma levels of clozapine and clozapine N-oxide (the steady-state trough of clozapine stayed around 220–250 ng/mL and never exceeded 260 ng/mL; the therapeutic “window” most guidelines now recommend is ≥350 ng/mL for clozapine and ≈10 ng/mL throughout treatment—roughly one-quarter of the CNO seen in the early-responder group). In contrast, plasma levels of N-desmethylclozapine were even higher than those of clozapine (1-year fixed dose of 600 mg/day; throughout the entire follow-up, the metabolite hovered around 280–320 ng/mL, always 60–70 ng/mL higher than the parent drug (which never climbed above ~260 ng/mL)). That predominance of the N-demethylation pathway is what Fabrazzo singled out as characteristic of the treatment-resistant subgroup. Although this was not the aim, one of the questions remaining from this study is whether it is possible to predict early response to clozapine treatment by looking at clozapine plasma levels [40].

#### 3.8.2. Serum Levels of Neurotrophins and Glutamate

The possibility that serum levels of neurotrophins and glutamate may provide clues as to how patients respond to clozapine treatment is also being explored [41].

Neurotrophins are proteins involved in the survival, development, and function of nerve cells. Measuring serum neurotrophin levels could be an indicator of neuronal function and provide information about the state of the nervous system. Glutamate is an excitatory neurotransmitter, and its levels may influence neuronal activity. Glutamate levels could provide information about neurotransmitter balance and synaptic activity [42].

Unlike treatment-resistant patients, clozapine responders had higher serum glutamate levels and brain-derived neurotrophic factor (BDNF)—a neurotrophin essential for the survival and function of neurons; vascular endothelial growth factor (VEGF)—involved in the formation and maintenance of blood vessels; neurotrophic growth factor (NGF)—another neurotrophin essential for the development and function of neurons; and glial-derived neurotrophic factor (GDNF)—a neurotrophin that exerts effects on neurons and glial cells and may have roles in the protection and survival of neurons [41].

The results give us hope for a biomarker of clozapine response, but these need to be tested in future clinical research.

#### 3.8.3. Morphometry

Individual brain morphometry in the first episode of schizophrenia could be a tool to help specialists predict prognosis and prescribe appropriate antipsychotic treatment. A recent study [43] shows that grey matter volume, especially in areas such as the orbitofrontal cortex, parietal cortex, temporal cortex, pallidum, and amygdala, could serve as an initial indicator of symptom progression and response to antipsychotic treatment, and thus is an indicator of the choice of the right antipsychotic.

#### 3.8.4. Genetic Testing

If we discussed above polygenic risk scoring, then genetic testing should not be overlooked in this equation.

Although genome-wide studies now implicate hundreds of loci, convergent functional studies keep DISC1, COMT, mono-amine-oxidase-A/B (MAO-A/B), GAD67 (GAD1), and NRG1 as possible triggers for schizophrenia because they link neurodevelopmental liability to the very transmitter and circuit motifs that clozapine ultimately modulates [44].

Post-GWAS fine-mapping still points to rare loss-of-function or mis-splicing variants that disturb cortical neurogenesis, synaptic plasticity, and striatal gating. A 2018 model lacking exons 2–3 reproduced perseverative/compulsive behaviours and learning deficits; c-Fos mapping localized hyper-activity to the dorsomedial striatum (DMS) [45]. In the same study, four-week oral clozapine (10 mg/kg) normalized DMS c-Fos and rescued both cognitive and repetitive-behaviour phenotypes—direct evidence that DISC1-driven circuit noise is pharmacologically tractable to clozapine’s multi-receptor profile.

The functional variant of the COMT gene Val158Met (rs4680) remains the most replicated dopaminergic modifier of prefrontal efficiency. Meta-analytic work since 2015 confirms modest risk enrichment of the Val allele in psychosis cohorts, particularly when stress or cannabis exposure is present [46]. In the same study in Bosia on 128 Italian patients, COMT Val/Val homozygotes showed the greatest improvement on the PANSS-Negative sub-scale after 12 weeks of clozapine, whereas Met carriers improved less [46]. In an Indian cohort of 93 treatment-resistant cases, COMT Met together with an intronic DRD4 120 bp duplication doubled the odds of response (BPRS ≤ 35) compared with either variant alone, supporting interactive dopaminergic control of clozapine efficacy [47]. Diminished expression of GAD67 in the cerebral cortex—with the attendant shortfall in GABA production—remains one of the most reproducible molecular abnormalities reported in schizophrenia autopsy studies. In a 2024 study, the conditional deletion of Zfp804a (a transcriptional regulator upstream of GAD1) precipitated a sharp drop in GAD67, disrupted the glutamate-to-GABA equilibrium, and produced a comprehensive behavioural phenotype characterized by hyperlocomotion, impaired prepulse inhibition, and working memory deficits [48]. Fourteen-day low-dose clozapine normalized both the GAD67 deficit and the behavioural profile in those Zfp804a-deficient mice, corroborating earlier epigenetic studies that showed that clozapine (but not haloperidol) drives promoter demethylation at GAD1. These convergent animal data explain why clozapine, uniquely among antipsychotics, can dampen cortical hyper-glutamatergia and improve cognitive subdomains in a subset of patients.

Rare coding variants in NRG1 and its receptor ERBB4 modulate interneuron maturation and excitatory–inhibitory balance. Deleting neuronal nitric oxide synthase (nNOS) specifically in Erbb4-positive interneurons (2024) produced the canonical triad—hyperlocomotion, sensorimotor-gating failure, and working memory loss [49]. All these deficits were fully reversed by clozapine (5 mg/kg for 10 days), again highlighting the drug’s ability to rectify interneuron dysfunction downstream of NRG1-ErbB signalling. In humans, a 2017 case–control study showed a selective upregulation of peripheral NRG1 EGFα/β transcripts in clozapine-treated patients, suggesting the pathway remains engaged under long-term therapy [50]. Although mRNA levels were not correlated with plasma drug concentrations, they did inversely relate to age of onset, arguing that NRG1 dys-expression marks a biologically distinct treatment-resistant subgroup.

Three biotransformation routes dominate clozapine disposition. N-demethylation to the active metabolite norclozapine is catalyzed above all by cytochrome P450 1A2 (CYP1A2), with contributory roles for CYP3A4, CYP2C19, and CYP2D6. N-oxidation to the inactive clozapine N-oxide proceeds primarily via flavin-containing mono-oxygenase-3 (FMO3), while the glucuronidation of both parent drug and norclozapine is mediated by uridine diphosphate-glucuronosyltransferases UGT1A4, UGT1A1, and UGT2B10. A genome-wide analysis of more than 10,000 routine TDM samples first linked common variation at CYP1A1/1A2, CYP2C18/CYP2C19, FMO3, and UGT1A4 to steady-state concentrations, explaining up to 9% of exposure variance in multivariate models [51]. Subsequent candidate gene studies have refined the functional relevance of individual alleles and their ethnic distributions.

CYP1A2 activity is highly inducible and modulated by several functional promoter variants. The 1F allele (rs762551, −163C > A) augments transcription in response to aryl-hydrocarbon receptor ligands such as polycyclic hydrocarbons in tobacco smoke. Among Tunisian in-patients, the CC genotype doubled the concentration-to-dose (C/D) ratio relative to AA homozygotes and accounted for almost one-quarter of inter-subject variability [52]. Conversely, the hypomorphic 1C allele (rs2069514), prevalent in East Asians, predisposes to dose-related adverse effects; Mexican carriers who also consumed alcohol reported significantly more neurological side-effects than non-carriers [53]. A loss-of-function 6 allele (rs72547516) is largely confined to Europeans, occurring at a frequency of about 1%. The genome-wide lead SNP upstream of CYP1A2 tags a haplotype in strong linkage disequilibrium with 1F and corresponds to a ≈50 mg/day difference in dose requirement per minor allele [51].

Importantly, genotype alone incompletely predicts the phenotype because environmental inducers (“phenoconversion”) superimpose on inherited activity. A Canadian cohort illustrated that reduced clozapine/norclozapine ratios and blunted working memory gains emerged only in smokers who were 1F carriers, not in non-smokers of the same genotype [54]. Allele frequencies underline the need for ancestry-adjusted titration: 1F is found in roughly 60% of Europeans, whereas 1C reaches 12% in East Asians but is rare elsewhere.

Although CYP2D6 contributes less than 15% to intrinsic clearance, its activity score modulates trough levels when smoking status and concomitant inhibitors are controlled. A longitudinal Korean study identified two intronic variants, rs28371726 and rs202102799, whose minor alleles increased trough concentrations by approximately 25% per copy after correction for dose, body mass index, and time [55]. In line with global allele frequency maps, poor metaboliser phenotypes cluster in Europeans (~8%), while ultrarapid metabolisers—usually conferred by gene duplications—are over-represented in North-East Africans.

CYP2C19 variation exerts subtler but measurable effects. Brazilian investigators showed that the gain-of-function *17 allele lowered Brief Psychiatric Rating Scale scores at any given dose, whereas *2/*2 homozygotes achieved therapeutic concentrations with about 100 mg/day less clozapine [56]. The 17 allele is present in nearly one-fifth of Europeans yet remains uncommon in East Asians, foreshadowing ethnicity-sensitive nomograms.

Polymorphisms in CYP3A4 seldom reach significance in clinical cohorts, but a 7q22 locus encompassing CYP3A4/CYP3A43 attained genome-wide association with clozapine N-oxide exposure, albeit with small effect sizes [51]. For FMO3, the missense variant rs2266782 (E158K), carried by nearly one in five East Asians, was associated with a 15% rise in the CNO/clozapine ratio in a Japanese study; however, no therapeutic repercussions have yet been demonstrated and the signal failed conventional genome-wide thresholds in European datasets [57].

Glucuronidation variants primarily influence norclozapine elimination. In the same Korean cohort that dissected P450 effects, UGT1A4 rs2011404 and UGT1A1 rs4148323 independently predicted norclozapine troughs and contributed to total exposure when integrated with CYP2D6 activity and anthropometric covariates [55]. The rs4148323 (UGT1A16) variant is virtually absent in Europeans but reaches frequencies of 16% in East Asians, providing a mechanistic explanation for the higher norclozapine levels typical of Chinese TDM series.

The convergent evidence suggests that early therapeutic drug monitoring remains indispensable; yet when a trough concentration appears discordant with the dose, targeted genotyping can refine clinical decisions. East Asian patients, enriched for CYP1A21C and UGT1A16, often require lower starting doses and careful monitoring for clozapine N-oxide accumulation. European male smokers harbouring CYP1A2*1F or CYP2D6 poor metaboliser alleles may need higher doses or split dosing to overcome inducible clearance, whereas North African ultrarapid metabolisers may benefit from early copy number testing if sub-therapeutic levels persist despite high doses.

Importantly, phenoconverting factors—strong CYP1A2 inhibitors (e.g., fluvoxamine, ciprofloxacin) or inducers like smoking, caffeine intake, omeprazole therapy, oral contraceptives (weak CYP1A2/CYP2C19 inhibition by estrogen), valproate co-medication, body mass, and ageing—still explain more exposure variance than genomics alone in head-to-head models [55,57]. They can halve or double clozapine clearance, respectively, or can be linked to a two-fold rise in clozapine levels [58]. Consequently, the genotype should complement, not replace, systematic TDM and assessment of modifiable covariates.

**Figure 2:**
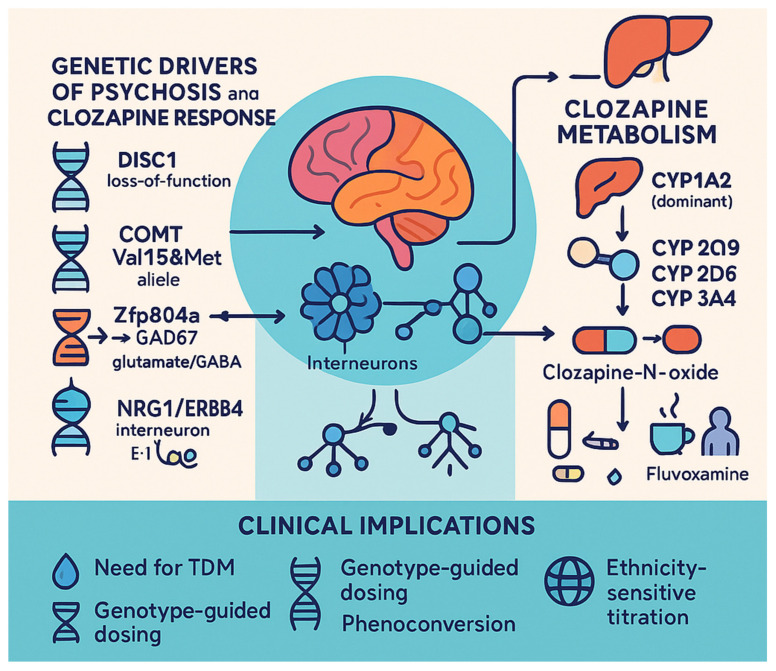
Clozapine pharmacogenetics and neurobiologics.

Dose adjustment intervals should respect the typical 8–16 h half-life, meaning plasma steady state is reached after roughly four days; however, clinicians must anticipate much longer tails in slow metabolizers or in the presence of CYP1A2 inhibition. Because CYP1A2 accounts for the majority of clearance, any change in smoking status, infection (cytokine-mediated CYP1A2 suppression), or co-prescribed inhibitors/inducers warrants repeat therapeutic drug monitoring. A 2025 update of the Clinical Pharmacogenetics Implementation Consortium and National Center for Biotechnology Information on Clozapine and CYP genotype monograph confirms CYP1A2 as the main determinant of dosing requirements, followed by CYP2C19 and CYP3A4; CYP2D6 plays a small, context-dependent role [59]. In Figure 2, we present the above-mentioned pharmacogenetics.

However, a potential genetic etiology subdivision of the disorder is also under discussion [60]. These are schizophrenia patients with chromosomal deletion 22q11.2. The latter, according to the source indicated, have a good response to clozapine treatment compared to idiopathic patients.

#### 3.8.5. Identifying Persistent Negative Symptoms

A different perspective is brought to the table by recent research that focuses on persistent negative symptoms (PNSs) in the first psychotic episode [61]. These are negative symptoms that persist in the absence of depression and pyramidal symptoms. Clozapine, in the research design, is used in the last stage only if the negative symptoms have not been improved by the antipsychotic medication given previously. But the response to the active substance in patients with persistent negative symptoms was poor. Their presence and persistence were attributed to a poor prognosis in terms of psychosocial functioning in patients with schizophrenia. Nevertheless, the need to clearly demarcate persistent negative symptoms (PNSs) of depression and extrapyramidal symptoms from the onset of the first psychotic episode is noted. In this way, it will be possible to predict the response to treatment with certain antipsychotics and to approach an appropriate and effective treatment scheme. In the next table (Table 3), we summarize the above-mentioned findings.

### 3.9. Side Effects

Without question, clozapine is one of the most effective atypical antipsychotics. As we have already pointed out, there is a well-founded fear of prescribing clozapine for the first manifestations of schizophrenia. This reluctance is largely due to the possibility of adverse reactions. Fortunately, this is being improved by testing, in parallel with the administration of antipsychotics, various active substances which reduce or even stop the undesirable effects.

#### 3.9.1. Cardiotoxicity of Clozapine

Although it is considered the gold standard treatment, in patients with treatment-resistant schizophrenia, clozapine has some side effects. These include cardiac ones [62]. Impaired left ventricular (LV) systolic and diastolic function has been observed. In addition, early exposure to clozapine in moderate doses may lead to changes in LV function. Although these are not clinically significant, in the long term, they could cause cardiac problems.

Understanding the potential for cardiotoxicity may provide the solution to developing treatment regimens that limit the dysfunction caused by the active substance.

#### 3.9.2. Metabolic Abnormalities

Another adverse reaction of clozapine is hyperglycemia [63]. This can cause acute or chronic reactions and can even lead to death in patients on antipsychotic treatment. The cause of the occurrence of hyperglycaemia appears to be due to the effect of the active substance on the blockade of H1, M1, M3, and 5-HT2C receptors that generate insulin resistance.

#### 3.9.3. Clozapine-Induced Hypersalivation (CIH)

One of the most common adverse effects is hypersalivation, and in order to combat this effect and reduce the worsening of cognitive deficits caused by hyoscine hydrobromide, glycopyrrolate administration has been studied [64] in patients developing ICH. Although it reduces hypersalivation, hyoscine hydrobromide generates cognitive deficits that hinder the psychopharmacological potential of clozapine. Compared to hyoscine hydrobromide, glycopyrrolate had a good effect on clozapine-induced hypersalivation and minimal reactions to cognitive deficits.

#### 3.9.4. Blood Disorders

In some cases, patients with schizophrenia on clozapine develop agranulocytosis [10], another undesirable and quite serious effect, given its lethal potential.

A limitation in clozapine administration in the African American population in the United States has also been identified due to the lower absolute neutrophil count (ANC) in this reference group [65]. They are twice as likely as the European American population to develop neutropenia. A polymorphism in the Duffy Antigen Receptor Chemokine (DARC) gene is responsible for this ethnic differentiation. Thus, clozapine treatment is not the first treatment option for AA patients with treatment-resistant schizophrenia.

#### 3.9.5. Weight Gain

One of the general problems faced by patients on clozapine is weight gain. To help reduce these unwanted side effects, betahistine has been tried with very good results [66]. Weight gain caused by antipsychotics has been attributed precisely to the specific effect of clozapine as a histaminergic antagonist and, in particular, a potent H1 receptor antagonist. Betahistine acts as a histamine H1 receptor agonist and H3 receptor antagonist, thereby enhancing histaminergic transmission.

In summary, Table 4 presents the major adverse effects of clozapine and mitigation strategies.

#### 3.9.6. Clozapine in Pregnancy and the Pediatric Population

Particular care should be taken when we are discussing pregnancy. Clozapine is not first-line in pregnancy; however, discontinuation may pose a greater danger than continuation for some women. If maintained, aim for the minimum effective dose, screen early for gestational diabetes, and check the neonate’s white cell count at birth.

A nested case–control study from the Australian National Register of Antipsychotic Medication in Pregnancy (NRAMP) reported higher rates of first-trimester miscarriage, gestational diabetes, and lower birth weight in 14 clozapine-exposed pregnancies when compared with quetiapine users and unmedicated controls [67]. By contrast, a systematic review that included only two low-quality studies concluded that no consistent signal of major malformation or obstetric complication has yet emerged, apart from a possible increase in low birth weight [68]. At the same time, global pharmacovigilance data drawn from VigiBase (22,956 clozapine reports) showed no excess of congenital malformation compared with other antipsychotics, but the authors caution that spontaneous reports cannot quantify absolute risk [69]. Consistent with a Danish cross-specialty guideline, clozapine is therefore not contraindicated in pregnancy but should be continued only when the mother is truly treatment-resistant, with intensified obstetric and metabolic monitoring [70].

When we are talking about the pediatric and adolescent use of clozapine, evidence remains largely limited to small observational studies. Clozapine can be highly effective in adolescents with genuine treatment resistance, but its narrow therapeutic window and rare cardiotoxicity demand adult-level monitoring. In pre-pubertal children, evidence is still insufficient. In a Turkish multi-centre series (*n* = 69) on adolescents with treatment-resistant schizophrenia, clozapine achieved symptom reductions comparable to electro-convulsive therapy and showed superior improvement in negative symptoms [71]. One new case of myocarditis reported in 2024 in a 15-year-old child underscores that the adult cardiotoxicity profile extends to youth [72]. The first systematic review dedicated to children identified 11 mainly open-label trials; clozapine consistently reduced severe aggression and self-injury in neuro-developmental and psychotic disorders, with effect sizes ≥ 1.0 on behaviour scales [73]. The main issue with this study is that the samples are small. The use of clozapine can be recommended only after two antipsychotic failures and only in settings able to provide weekly blood counts and cardiac surveillance.

#### 3.9.7. Gender Differences in Clozapine Response in Males Versus Females

Over the past decade, pharmaco-epidemiological datasets have converged to a consistent pattern: women with treatment-resistant schizophrenia are significantly less likely than men to receive clozapine, and when they do, the drug is usually introduced later. A pan-European registry of 1402 patients calculated that being of the female sex reduced the odds of ever being prescribed clozapine by one-third after controlling for age and illness severity [74]. An independent 30-year Italian cohort replicated both the lower initiation rate and shorter treatment persistence in women, suggesting that clinician caution about fertility, pregnancy, and weight gain, rather than evidence of inferior efficacy, drives this disparity [75].

Pharmacokinetic studies align with these prescribing observations. Three independent therapeutic drug monitoring (TDM) programmes report that dose-normalized trough concentrations are about 20–25% higher in females, even after adjustment for smoking, body weight, and age [76,77]. A population PK model built from 48,000 plasma samples predicts that women require roughly 22% lower daily doses than otherwise comparable men to reach the canonical 350 ng/mL therapeutic target [77]. Ancillary laboratory work further links female sex to higher body mass index, glucose, and clozapine exposure, suggesting a genuine metabolic difference rather than underdosing in males [78].

Whether these pharmacokinetic differences translate into better clinical outcomes remains uncertain. A 2019 systematic review found the evidence base too sparse to draw firm conclusions on sex-specific efficacy [79]. Subsequent primary studies have been mixed: an Italian tolerability trial (*n* = 147) detected greater improvement in negative symptoms among women, whereas a South Asian cohort reported no sex effect on response once adherence and dose were taken into account [80,81]. Taken together, the literature points at most to a modest female advantage that is easily masked by confounders such as earlier illness onset and heavier smoking in men.

Adverse event profiles, by contrast, show clear qualitative differences. An analysis of more than 3000 patients in the CLOZIN consortium identified tachycardia, postural hypotension, and constipation as female-predominant side-effects, whereas hypertension and dyslipidemia clustered in men [82]. The 2019 systematic review reached similar conclusions, adding that weight gain, diabetes, and laxative use are more common in women [79]. Many of these events correlate more strongly with plasma concentration than with prescribed dose, reinforcing the pharmacokinetic sex gap.

Clinical implications are straightforward. First, titration algorithms should subtract roughly one-fifth from the “standard” male dose for non-smoking women, with repeat TDM after any change in smoking status. Second, monitoring needs to be sex-sensitive: women warrant closer surveillance for autonomic and metabolic toxicity, whereas men, who tend to receive higher doses to reach target levels, require aggressive cardiovascular risk management. Finally, because efficacy appears comparable (if not slightly better) in women, the documented under-prescription constitutes an avoidable treatment gap that should be addressed in future guideline updates.

## 4. Discussion

Although superior to other antipsychotics, clozapine appears not to be used to its full potential. Adverse reactions are perhaps the ones that cause reluctance when it comes to taking the active substance. There is significant interest in reducing unwanted side effects. An example is the administration of betahistine to reduce the risk of obesity or the replacement of hyoscine hydrobromide with glycopyrrolate, which eliminated the cognitive deficits produced by hyoscine hydrobromide and had a very good effect on clozapine-induced hypersalivation. Also, understanding the potential for cardiotoxicity may provide solutions for developing treatment regimens that limit clozapine-induced dysfunction.

Furthermore, attempts are being made to increase the effectiveness of clozapine treatment by augmenting it with antipsychotics such as amisulpride and paliperidone, anti-inflammatory drugs such as minocycline, and other active substances such as N-acetylcysteine (NAC) or memantine. Augmentation with another active substance may have stronger effects in reducing negative, positive, and cognitive symptoms.

Perhaps the most important aspect is the predictability of treatment. This would be a game changer in psychiatric practice and in the administration of new treatment regimens. In this direction, there are significant indications of the possibility of early prediction of response to clozapine treatment by looking at reduced plasma levels of clozapine N-oxide and increased plasma levels of N-desmethylclozapine; higher serum levels of glutamate and neurotrophins could serve as potential biomarkers of clozapine response. Added to these are individual brain morphometry, genetic testing, especially in schizophrenia patients with a chromosomal deletion 22q11.2 (these having a good response to clozapine treatment) polygenic risk score, as a potential aid in the early prediction of treatment response and the determination of an effective treatment regimen.

## Figures and Tables

**Table 1:** Clozapine mechanisms of action and targets.

Receptor/Target	Clozapine Action	Effect/Implication	Reference
Dopamine receptors (D1–D5; high affinity D4)	Competitive antagonist (low-affinity, fast-off at D2; higher affinity at D4)	Modulation of cognitive, motor, and behavioural functions and underlies antipsychotic efficacy with fewer extrapyramidal effects	[18,19]
Serotonin receptors (5-HT_2_A/_2_C)	Antagonist	Augments dopamine modulation in cortex/limbic areas, contributing to efficacy for negative symptoms	[19]
Muscarinic receptors (M1, M2, M3, M5)	Antagonist	Responsible for anticholinergic side effects and pro-cognitive metabolite activity	[19,20]
Histamine H_1_ receptor	Antagonist	May cause sedation and weight gain	[20]
Alpha-1 adrenergic receptor	Antagonist	Impacts vascular tone, may cause orthostatic hypotension	[20]
NMDA receptor	Functional (indirect) partial agonism via metabolite NDMC; clinical interest in glycine-site augmentation	Potentially reduces dopaminergic hyperactivity	[16]
mTOR pathway (ribosomal protein S6)	Regulatory modulation of protein synthesis/neuroplasticity	Impacts protein synthesis, cell survival, neuroplasticity	[19]
Kynurenine 3-Monooxygenase (KMO)	Inhibition	Reduces dopamine neuron hyperactivity via NMDA/α7 nicotinic modulation	[15]
Regional cerebral blood flow	Increase (esp. right inferior frontal gyrus and other cortical areas)	Improved perfusion in specific brain areas to reduced impulsivity/aggression and potential structural recovery	[11]

**Table 2:** Clozapine augmentation strategies for treatment-resistant schizophrenia.

Augmentation Agent/Therapy	Mechanism/Role	Effect	Considerations
Amisulpride	D2/D3 antagonist	Improves negative and cognitive symptoms	Risk of cardiac side effects
Paliperidone	D2 antagonist, serotonin antagonist	Effective, safer compared to amisulpride	Requires more randomized trials
Minocycline	Anti-inflammatory, neuroprotective	Reduces inflammation, well-tolerated	Small sample studies, needs replication
N-Acetylcysteine (NAC)	Antioxidant, glutathione precursor	Minimal improvement in schizophrenia symptoms	Limited efficacy observed
Memantine	NMDA receptor antagonist	Improves memory, executive function, negative symptoms	Promising, well-tolerated
Repetitive Transcranial Magnetic Stimulation (rTMS)	Non-invasive brain stimulation	Enhances antipsychotic efficacy, reduces psychotic symptoms	Requires more research
Electroconvulsive Therapy (ECT)	Electrical stimulation therapy	Effective in treatment-resistant cases	Long-term efficacy needs more studies

**Table 3:** Predictors of clozapine treatment response.

Predictive Factor	Details/Indicators	Relevance to Response
Plasma Levels	Lower clozapine and clozapine N-oxide; higher N-desmethylclozapine in non-responders	May predict poor response
Serum Neurotrophins and Glutamate	Higher BDNF, VEGF, NGF, GDNF, and glutamate levels in responders	Possible biomarkers
Brain Morphometry	Grey matter volume in PFC, parietal cortex, amygdala	May predict symptom progression and drug response
Polygenic Risk Score	High PRS linked to clozapine prescription	May assist in early intervention strategies
Genetic Testing	Specific polymorphisms in DISC1, COMT, MAO-A/B, GAD67, NRG1; 22q11.2 deletion	Potential influence on responsiveness
Persistent Negative Symptoms (PNSs)	Poor response when present	Early identification aids treatment planning

**Table 4:** Major adverse effects of clozapine and mitigation strategies.

Adverse Effect	Cause/Mechanism	Mitigation Strategy
Cardiotoxicity	Impaired left ventricular function	Monitoring, dose adjustment
Hyperglycemia	Blockade of H1, M1, M3, 5-HT2C receptors	Metabolic monitoring, insulin-sensitizing agents
Hypersalivation (CIH)	Muscarinic receptor involvement	Glycopyrrolate preferred over hyoscine hydrobromide
Agranulocytosis/Neutropenia	Possibly related to genetic polymorphisms (DARC gene in AA population)	Monitoring ANC, cautious use in at-risk groups
Weight Gain	Strong H1 receptor antagonism	Betahistine (H1 agonist, H3 antagonist) administration
